# Double-Color-Image Compression-Encryption Algorithm Based on Quaternion Multiple Parameter DFrAT and Feature Fusion with Preferable Restoration Quality

**DOI:** 10.3390/e24070941

**Published:** 2022-07-06

**Authors:** Meihua Xiao, Ruixiao Tan, Huosheng Ye, Lihua Gong, Zhiliang Zhu

**Affiliations:** 1 School of Software, East China Jiaotong University, Nanchang 330013, China; xiaomh@ecjtu.edu.cn (M.X.); trxhdjd@163.com (R.T.); 2 Department of Electronic Information Engineering, Nanchang University, Nanchang 330031, China; yhsncu@163.com (H.Y.); ncuglh@163.com (L.G.); 3 The State Key Laboratory of Computer Science, Institute of Software, Chinese Academy of Sciences, Beijing 100190, China

**Keywords:** double-color-image encryption, quaternion, non-adjacent coupled map lattices, random pixel insertion

## Abstract

To achieve multiple color images encryption, a secure double-color-image encryption algorithm is designed based on the quaternion multiple parameter discrete fractional angular transform (QMPDFrAT), a nonlinear operation and a plaintext-related joint permutation-diffusion mechanism. QMPDFrAT is first defined and then applied to encrypt multiple color images. In the designed algorithm, the low-frequency and high-frequency sub-bands of the three color components of each plaintext image are obtained by two-dimensional discrete wavelet transform. Then, the high-frequency sub-bands are further made sparse and the main features of these sub-bands are extracted by a Zigzag scan. Subsequently, all the low-frequency sub-bands and high-frequency fusion images are represented as three quaternion signals, which are modulated by the proposed QMPDFrAT with three quaternion random phase masks, respectively. The spherical transform, as a nonlinear operation, is followed to nonlinearly make the three transform results interact. For better security, a joint permutation-diffusion mechanism based on plaintext-related random pixel insertion is performed on the three intermediate outputs to yield the final encryption image. Compared with many similar color image compression-encryption schemes, the proposed algorithm can encrypt double-color-image with higher quality of image reconstruction. Numerical simulation results demonstrate that the proposed double-color-image encryption algorithm is feasibility and achieves high security.

## 1. Introduction

In recent years, secure transmission of color images has attracted widespread attention. Due to the intrinsic features of images, such as strong correlation between adjacent pixels, large storage capacity, and high redundancy, the traditional textual encryption algorithms, such as DES (Data Encryption Standard) and AES (Advanced Encryption Standard), are not suitable for image encryption [1]. To ensure the confidentiality of the private image information, a great deal of color image encryption algorithms have been presented with various technologies. Among these algorithms, chaotic systems have been widely adopted in image encryption owing to its excellent properties. For instance, Pak et al. introduced a simple and effective method of generating a new chaotic sequence according to the differences in the output sequences of two existing one-dimensional (1D) chaotic maps [2]. Based on the obtained sequences, a linear-nonlinear-linear encryption structure of this cryptographic system was designed to improve security. Similarly, a color image encryption scheme based on a new combination chaotic system was proposed [3]. Nevertheless, the chaotic dynamic properties degrade rapidly in computer realization with finite computation precision [4]. To overcome this problem, a spatiotemporal chaotic system, i.e., the non-adjacent coupled map lattices (NCML) was developed to alleviate the degradation of low-dimensional chaos map [5]. Subsequently, a series of image encryption algorithms were proposed based on the spatiotemporal chaotic system [6,7]. Moreover, for better security and larger key space, the high-dimensional chaotic systems have been increasingly employed to design the image encryption schemes [8,9,10,11]. In ref. [8], a novel 3D chaotic map obtained by coupling the piecewise and logistic map is implemented to improve the performance of cryptosystem. Tong et al. proposed a fast image encryption scheme based on a new 4D chaotic system [9]. In addition, to enhance the security and complexity of the cryptosystems, chaos-based encryption schemes were combined with other methods, such as deoxyribonucleic acid [12], cellular automata [13], fuzzy cellular neural network [14], and so on.

However, the permutation and the diffusion processes in these encryption schemes were both performed in the spatial domain, which may reduce the security of cryptosystems to some extent. Inspired by some excellent mathematical properties of transform techniques, many researchers have attempted to devise transform-based image encryption algorithms, where the plaintext image is encrypted in the transform domain and the pixel values can be retrieved through a reverse process. To the best of our knowledge, there are few investigations on double-color-image compression and encryption algorithms. In light of this situation and the above discussions, a new double-color-image compression and encryption algorithm based on QMPDFrAT and a joint permutation-diffusion mechanism are designed. The main contributions of the proposed algorithm are as follows:

(1) Multiple parameter discrete fractional angular transform (MPDFrAT) is generalized to quaternion MPDFrAT. The analysis shows its advantages in image encryption. Then, the sub-bands of the original images can be encrypted with the proposed QMPDFrAT in a parallel way.
(2) The deficiency caused by the linear transform system is eliminated by nonlinear transform, i.e., spherical transform.
(3) A joint permutation-diffusion mechanism with plaintext-related random pixel insertion is designed to ensure the proposed cryptosystem could counteract the powerful chosen-plaintext attack and improve the efficiency of the cryptosystem.
(4) The effect of different components of the high-frequency sub-bands on the quality of the decryption image is discussed and a more reasonable feature fusion method of the high-frequency part is implemented by combining DWT with Zigzag operation. Consequently, the proposed image encryption algorithm could achieve higher quality of the decryption images than that of the typical image compression and encryption algorithms.

The rest of this paper is arranged as follows. In Section 2, some related works are discussed. In Section 3, some fundamental tools including the NCML system, quaternion algebra, and MPDFrAT are reviewed. The QMPDFrAT is defined and analyzed in Section 4. The details of the proposed double-color-image encryption algorithm are described in Section 5. In Section 6, simulation results and security evaluations are provided. Brief conclusions are given in Section 7.

## 2. Related Works

Based on the gyrator transform, Chen et al. proposed an asymmetric optical cryptosystem for the color image [15]. Xiong et al. designed an optical color image scheme based on fractional Fourier transform and two-step phase-shifting interferometry [16]. Nevertheless, these schemes processed each color channel separately and failed to capture the inherent correlation among three color channels. To deal with the three color channels parallelly, many encryption methods have been investigated with the quaternion-based transforms [17,18,19]. However, the outputs of these aforementioned transform-based algorithms are complex values and the size of encryption results or private keys exceed that of the original images, which may make the transmission and storage of encryption image and private keys inconvenient. To overcome this insufficiency, Zhou et al. devised a nonlinear color image encryption algorithm based on reality-preserving fractional Mellin transform, where the final output was real-value encrypted image [20]. Motivated by this work, many other reality-preserving transforms were defined to encrypt the color image [21,22,23]. To enhance the capacity of the cryptosystem, multiple color image encryptions have attracted increasing attention [24,25,26]. For example, Shao et al. designed a multiple color images encryption framework, in which the multiple color images were encrypted into phase-only function with phase retrieval algorithm under quaternion representation [24]. In many practical applications, for the facility of transmission of ciphertext image, it is necessary to realize simultaneous image compression and encryption. As a novel signal sampling-reconstruction technique, compressive sensing (CS) has been widely employed to solve this problem [27,28,29,30]. For instance, Chen et al. put forward an asymmetric color cryptographic system, in which not only the low-frequency but also the CS-based compressed high-frequency part of the original image were encrypted in the discrete fractional random transform domain [29]. To enhance encryption efficiency, Zhang et al. investigated an efficient color image encryption approach based on CS and fractional Fourier transform, where the measurement matrices exploited in CS were obtained by extending chaos-based low dimensional seed matrices with Kronecker product [30]. However, these transform-based color image compression-encryption schemes were only designed for single color image, which makes them unable to process batch images efficiently to a certain extent. Aiming at this problem, Han et al. suggested a double-color-image compression and encryption algorithm based on CS and self-adaptive random phase encoding [31]. However, in some special applications, the decryption time and the quality of decryption image are also of significance. Table 1 shows the decryption time and the PSNR values of the test image “Peppers” under different reconstruction algorithms with the same compression ratio. Unfortunately, the signal reconstruction takes too much time even though many efficient reconstruction algorithms including orthogonal matching pursuit (OMP) and smoothed norm have been proposed. In other words, the DWT-based compression method may be a good choice in real-time decryption applications.

## 3. Fundamental Knowledge

### 3.1. Non-Adjacent Coupled Map Lattices System

The non-adjacent coupled map lattices system is considered as the improved spatiotemporal chaotic system, which can generate pseudorandom sequences with stable chaotic properties [5]. The NCML considers L logistic maps coupled as

(1) $$x_{n+1}(\rho )=(1-\delta )f[x_{n}(\rho )]+\frac{\delta }{2}\left\{f[x_{n}(\upsilon )]+f[x_{n}(\omega )]\right\}$$

where f(x)=λx(1−x) is logistic map, δ is the coupling parameter (0≤δ≤1), n is the time index (n=1,2,3,…), and ρ, υ, ω are the lattices (1≤ρ,υ,ω≤L). The relations of ρ, υ, and ω can be obtained by Arnold cat map, i.e.,

(2) $$\left[\begin{array}{c} \upsilon \\ \omega \end{array}\right]=\left[\begin{array}{cc} 1 & 1\\ 1 & 2 \end{array}\right]\left[\begin{array}{c} \rho \\ \rho \end{array}\right]\mathrm{mod}(L)$$

### 3.2. Quaternion Representation of Multi-Image

Quaternions are hyper-complex numbers with four dimensions. A quaternion number Q is [32]

(3) Q=a+bi+cj+dk

where a, b, c, d are real numbers and i, j, k are three imaginary operators acting on the following rules.

(4) $$i^{2}=j^{2}=k^{2}=-1,\;ij=-ji=k,\;jk=-kj=i,\;ki=-ik=j$$

The modulus and the conjugate of a quaternion are respectively defined as

(5) $$\left|Q\right|=\sqrt{a^{2}+b^{2}+c^{2}+d^{2}},\;Q^{*}=a-bi-cj-dk$$

If the real part a is 0, then Q is called a pure quaternion. If the modulus |Q| equals to 1, then Q is called a unit quaternion. Based on the above theory, the quaternion representation of multi-image is [17]

(6) $$f_{Q}(x,y)=f_{1}(x,y)+f_{2}(x,y)i+f_{3}(x,y)j+f_{4}(x,y)k$$

where $f_{Q}(x,y)$ is a quaternion signal and $f_{1}(x,y)$, $f_{2}(x,y)$, $f_{3}(x,y)$, $f_{4}(x,y)$ are four image signals, respectively.

### 3.3. Multiple Parameter Discrete Fractional Angular Transform

Briefly, the definition of the discrete fractional angular transform (DFrAT) is introduced. The kernel matrix of the DFrAT is [33]

(7) $$R_{N}^{\alpha ,\theta }=V_{N}^{\theta }D_{N}^{\alpha }{\left(V_{N}^{\theta }\right)}^{T}$$

where $D_{N}^{\alpha }=\mathrm{diag}\left\{1,\exp\left(-2i\pi \alpha /M\right),\exp\left(-4i\pi \alpha /M\right),\ldots ,\exp\left(-2\left(N-1\right)i\pi \alpha /M\right)\right\}$ is a diagonal matrix, whose diagonal values are eigenvalues of the DFrAT, $V_{N}^{\theta }$ is an orthonormal matrix and consists of the eigenvectors of the DFrAT. $V_{N}^{\theta }$ can be obtained with a recurrence algorithm elaborated in [33].

Based on the DFrAT, a new multiple parameter discrete fractional angular transform (MPDFrAT) was presented [34]. For a 1D signal x(n) of size N×1, its αth order 1D MPDFrAT is [34]

(8) $$F_{M,\eta_{1}}^{\alpha ,\theta }\left[x\left(n\right)\right]=\sum_{l=0}^{M-1}C_{l}^{\alpha }\left(\eta_{1}\right)X_{l}\left[x\left(n\right)\right]$$

where M is an arbitrary positive integer, $\eta_{1}=(n_{0},n_{1},\ldots ,n_{M-1})\in Z^{M}$ is a random *M*-dimensional integer vector, l=0,1,2,…,M−1, $C_{l}^{\alpha }\left(\eta_{1}\right)$ denotes the weight coefficient given by

(9) $$C_{l}^{\alpha }\left(\eta_{1}\right)=\frac{1}{M}\sum_{k=0}^{M-1}\left\{\exp(-2\pi i/M)\left[\alpha \left(k+n_{k}M\right)-lk\right]\right\}$$

$X_{l}\left[x\left(n\right)\right]$ denotes 1D DFrAT with the angle θ and the fractional order 4l/M, i.e.,

(10) $$X_{l}\left[x\left(n\right)\right]=R_{N}^{4l/M,\theta }x\left(n\right)$$

where $R_{N}^{4l/M,\theta }$ denotes the kernel matrix of DFrAT and can be obtained with Equation (7).

In fact, the MPDFrAT has a similar form with the multiple parameter discrete fractional Fourier transform (MPDFrFT). The difference between MPDFrAT and MPDFrFT is the generation process of the eigenvector of the kernel matrix. To calculate the MPDFrAT of a discrete signal in an efficient way, one can utilize a discretization method [19] by eigen-decomposing MPDFrAT matrix ${℘}_{M,\eta_{1}}^{\alpha ,\theta }$ as

(11) $$\begin{array}{cc} {℘}_{M,\eta_{1}}^{\alpha ,\theta } & =V_{N}^{\theta }D_{N}^{\alpha }{\left(V_{N}^{\theta }\right)}^{T}\\ & =\sum_{t=0}^{N-1}\exp\left\{\left(-2\pi i/M\right)\left[\alpha \left(\mathrm{mod}\left(m_{t},M\right)+n_{\mathrm{mod}\left(m_{t},M\right)}M\right)\right]\right\}v_{m_{t}}v_{m_{t}}^{T}, \end{array}$$

where $m_{t}=t$, $m_{N-1}=N$ for even N while $m_{N-1}=N-1$ for odd N, mod(⋅) denotes the modulo operation, $v_{m_{t}}$ is eigenvector of DFrAT.

Then, one can rewrite the $\alpha^{\mathrm{th}}$ order 1D MPDFrAT of a signal x as an eigen-decomposition form, i.e.,

(12) $${ℑ}_{M,\eta_{1}}^{\alpha ,\theta }={℘}_{M,\eta_{1}}^{\alpha ,\theta }x$$

## 4. Quaternion Multiple Parameter Discrete Fractional Angular Transform

Enlighted by the idea of the definition for 1D MPDFrAT with eigen-decomposition form shown in Equation (11), one can define a new quaternion multiple parameter discrete fractional angular transform (QMPDFrAT) for quaternion signal. For a 1D quaternion signal $x_{q}=x_{r}+x_{i}i+x_{j}j+x_{k}k$, its left-side QMPDFrAT is defined as

(13) $$\Phi_{M,\eta_{1}}^{\mu ,\alpha ,\theta }={ℜ}_{M,\eta_{1}}^{\mu ,\alpha ,\theta }x_{q}$$

where

(14) $$\begin{array}{cc} {ℜ}_{M,\eta_{1}}^{\mu ,\alpha ,\theta } & =V_{N}^{\theta }D_{M}^{\mu ,\alpha }{\left(V_{N}^{\theta }\right)}^{T}\\ & =\sum_{t=0}^{N-1}\exp\left\{\left(-2\pi \mu /M\right)\left[\alpha \left(\mathrm{mod}\left(m_{t},M\right)+n_{\mathrm{mod}\left(m_{t},M\right)}M\right)\right]\right\}v_{m_{t}}v_{m_{t}}^{T} \end{array}$$

Equation (14) is similar to MPDFrAT matrix ${℘}_{M,\eta_{1}}^{\alpha ,\theta }$ in Equation (11) and complex number i is replaced by quaternion μ. Due to the anticommutation of the multiplication of quaternions shown in Equation (4), one can also define the right-side 1D QMPDFrAT by shifting the kernel matrix ${ℜ}_{M,\eta_{1}}^{\mu ,\alpha ,\theta }$ to the right-side of $x_{q}$, i.e.,

(15) $$\Phi^{\prime }{}_{M,\eta_{1}}^{\mu ,\alpha ,\theta }={\left(x_{q}\right)}^{T}{ℜ}_{M,\eta_{1}}^{\mu ,\alpha ,\theta }$$

Without loss of generality, the left-side 1D QMPDFrAT is exploited in this paper. In addition, to illustrate the feature of the proposed QMPDFrAT, a 1D quaternion signal of size 256 × 1 is transformed by using the fractional quaternion Fourier transform (FRQFT) [35], the quaternion discrete fractional random transform (QDFRNT) [36], the multiple-parameter fractional quaternion Fourier transform (MPFrQFT) [19], and the proposed QMPDFrAT. The comparison results are recorded in Table 2. For brevity, only the first imaginary parts of the input and output quaternion signal are drawn in Table 2. The complicated Hermite polynomials calculation for eigenvectors in the MPFrQFT and the Schmidt orthogonalization of a random matrix in the QDFRNT require relatively higher time. However, the eigenvectors in the QMPDFrAT can be obtained only by simple recurrences and thus the calculation speed is greatly improved. Furthermore, the proposed QMPDFrAT possesses the largest key space among these four quaternion transforms. Therefore, the proposed QMPDFrAT is a suitable tool for image encryption.

The 1D QMPDFrAT can be developed to the 2D one by calculating two 1D QMPDFrATs in the *x*-axis and the *y*-axis, respectively, i.e.,

(16) $$Y_{M_{1},M_{2},\eta_{1},\eta_{2}}^{\mu ,\alpha ,\beta ,\theta_{1},\theta_{2}}={ℜ}_{M_{1},\eta_{1}}^{\mu ,\alpha ,\theta_{1}}y_{q}{\left({ℜ}_{M_{2},\eta_{2}}^{\mu ,\beta ,\theta_{2}}\right)}^{T}$$

where $y_{q}=y_{r}+y_{i}i+y_{j}j+y_{k}k$ is a 2D quaternion signal.

## 5. Double-Color-Image Compression and Encryption Algorithm

**Figure 1:**
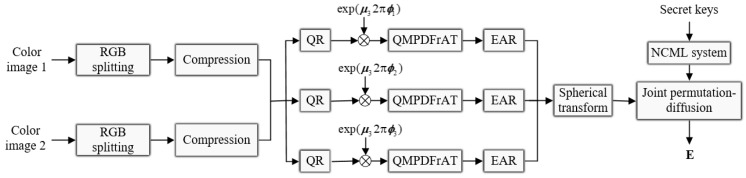
Double-color-image encryption algorithm.

The encryption process for the designed double-color-image encryption algorithm is shown in Figure 1. The main encryption processes include three stages: sparse representation of the color plaintext images, double-color-image encryption in the frequency domain under quaternion representation, and joint permutation-diffusion mechanism. The detailed steps are described as follows.

### 5.1. Compression Process

**Step 1**: Double-color plaintext images $C_{1}$ and $C_{2}$ of size W×H are converted into their red, green, and blue components which can be expressed as $R_{1}$, $G_{1}$, $B_{1}$, $R_{2}$, $G_{2}$, and $B_{2}$, respectively.

**Step 2**: The one level 2D DWT is performed on the six color components to obtain 24 image sub-bands of size $\frac{W}{2}\times \frac{H}{2}$, namely, $\left\{LL_{1}^{R_{1}},LH_{1}^{R_{1}},HL_{1}^{R_{1}},HH_{1}^{R_{1}}\right\}$ for $R_{1}$, $\left\{LL_{1}^{G_{1}},LH_{1}^{G_{1}},HL_{1}^{G_{1}},HH_{1}^{G_{1}}\right\}$ for $G_{1}$, $\left\{LL_{1}^{B_{1}},LH_{1}^{B_{1}},HL_{1}^{B_{1}},HH_{1}^{B_{1}}\right\}$ for $B_{1}$, $\left\{LL_{1}^{R_{2}},LH_{1}^{R_{2}},HL_{1}^{R_{2}},HH_{1}^{R_{2}}\right\}$ for $R_{2}$, $\left\{LL_{1}^{G_{2}},LH_{1}^{G_{2}},HL_{1}^{G_{2}},HH_{1}^{G_{2}}\right\}$ for $G_{2}$, $\left\{LL_{1}^{B_{2}},LH_{1}^{B_{2}},HL_{1}^{B_{2}},HH_{1}^{B_{2}}\right\}$ for $B_{2}$.

**Figure 2:**
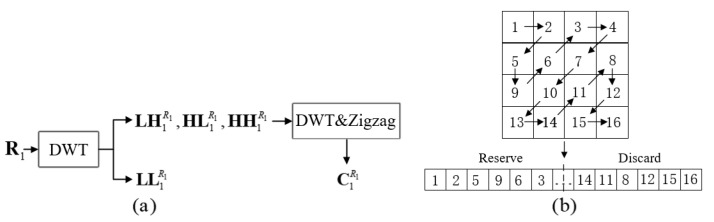
Compression process for $R_{1}$. (**a**) shows the whole compression process and (**b**) shows the schematic diagram of Zigzag operation in (**a**).

**Step 3**: Low-frequency parts of six spectra are chosen as the three imaginary parts of two quaternion signals $f_{Q_{1}}$ and $f_{Q_{3}}$. To improve the quality of image reconstruction and achieve small transmission load simultaneously, a new method for processing high-frequency parts of six spectra is designed as follows:

(1) The sub-bands $LH_{1}^{R_{1}}$, $HL_{1}^{R_{1}}$ and $HH_{1}^{R_{1}}$ of $R_{1}$ are transformed by the DWT and the interim results are scanned by the Zigzag operation [37] to obtain three 1D sequences with length $\frac{1}{4}WH$, respectively. The compression process for $R_{1}$ is drawn in Figure 2a.
(2) Each sequence is cut to acquire a new sequence with length ⌊(WH)/12⌋. Figure 2b shows the schematic diagram of Zigzag operation. The results after performing DWT on the high-frequency parts are scanned from the upper left corner to the lower right corner. This scan order can extract the main information of the high-frequency parts, which promises the preferable restoration quality as analyzed in Section 5.2.
(3) The above-mentioned three new sequences are constructed into one sequence. If necessary, the zero elements are filled in the right-most row of this reorganized sequence to ensure that the length of this sequence is (WH)/4. Then, this sequence is converted into a composite matrix $C^{R_{1}}$ of size $\frac{W}{2}\times \frac{H}{2}$.
(4) In a similar way, one can obtain five other composite matrices $C^{G_{1}}$, $C^{B_{1}}$, $C^{R_{2}}$, $C^{G_{2}}$, and $C^{B_{2}}$ from five high-frequency parts of $G_{1}$, $B_{1}$, $R_{2}$, $G_{2}$, and $B_{2}$, respectively.

### 5.2. Double-Color-Image Encryption under Quaternion Representation

**Step 1**: Quaternion representation (QR): the selected to-be-encrypted sub-bands are represented by quaternion algebra shown as follows

(17) $$\left\{ \begin{array}{l} f_{Q_{1}}=C^{R_{1}}+LL_{1}^{R_{1}}i+LL_{1}^{G_{1}}j+LL_{1}^{B_{1}}k\\ f_{Q_{2}}=C^{G_{1}}+C^{R_{2}}i+C^{G_{2}}j+C^{B_{2}}k\\ f_{Q_{3}}=C^{B_{1}}+LL_{1}^{R_{2}}i+LL_{1}^{G_{2}}j+LL_{1}^{B_{2}}k \end{array}\right.$$

**Step 2**: With the secret keys $M_{1}$, $M_{2}$, $\eta_{1}$, $\eta_{2}$, α, β, $\theta_{1}$, $\theta_{2}$, $\mu_{1}$, $\mu_{2}$, and $\mu_{3}$, three quaternion signals shown in Equation (17) are modulated by the proposed QMPDFrAT with three quaternion random phase masks, respectively.

(18) $$G_{i}={ℜ}_{M_{1},\eta_{1}}^{\mu_{1},\alpha ,\theta_{1}}\left[f_{Q_{i}}\exp\left(\mu_{3}2\pi \phi_{i}\right)\right]{\left({ℜ}_{M_{2},\eta_{2}}^{\mu_{2},\beta ,\theta_{2}}\right)}^{T}\left(i=1,2,3\right)$$

where phase mask $\phi_{i}(i=1,2,3)$ is a random matrix uniformly distributed in [0,1).

**Step 3**: The four parts of each quaternion signal $G_{i}$ are extracted and reorganized (EAR) to form a new matrix of size W×H.

(19) $$A_{i}=\left[\begin{array}{cc} R\left(G_{i}\right) & X\left(G_{i}\right)\\ Y\left(G_{i}\right) & Z\left(G_{i}\right) \end{array}\right]\left(i=1,2,3\right)$$

where $R\left(G_{i}\right)$, $X\left(G_{i}\right)$, $Y\left(G_{i}\right)$, and $Z\left(G_{i}\right)$ extract the real part and the three imaginary parts of the quaternion signal $G_{i}$, respectively.

**Figure 3:**
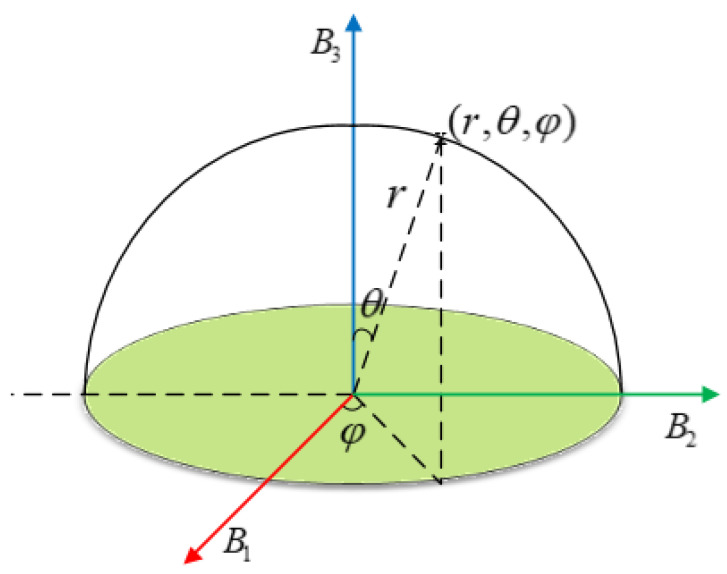
Rotation map on matrix $B_{i}(i=1,2,3)$ in spherical transform.

**Step 4**: To eliminate the deficiency caused by the inherent linearity of the QMPDFrAT, a nonlinear operation called spherical transform is followed to further hide the information obtained by matrices $A_{1}$, $A_{2}$, and $A_{3}$. For the convenience of decryption, the matrix $A_{i}\left(i=1,2,3\right)$ is first mapped to the matrix $B_{i}\left(i=1,2,3\right)$ whose values are greater than zero. As shown in Figure 3, the three matrices $B_{1}$, $B_{2}$, and $B_{3}$ can be regarded as the orthorhombic axis in the spherical coordinate domain. The mapping rules are

(20) $$\left\{ \begin{array}{c} r\left(u,v\right)=\sqrt{B_{1}{\left(u,v\right)}^{2}+B_{2}{\left(u,v\right)}^{2}+B_{3}{\left(u,v\right)}^{2}}\\ \theta \left(u,v\right)=\varepsilon \cdot \arccos\frac{B_{3}\left(u,v\right)}{r\left(u,v\right)}\\ \varphi \left(u,v\right)=\varepsilon \cdot \arctan\frac{B_{2}\left(u,v\right)}{B_{1}\left(u,v\right)} \end{array}\right.$$

where ε is a plaintext-related adjustment factor which can be calculated as

(21) $$\begin{array}{ll} \varepsilon & =\frac{\mathrm{sum}}{255^{3}}\\ & =\frac{1}{255^{3}}\sum_{i=1}^{W}\sum_{j=1}^{H}\left[R_{1}\left(i,j\right)+G_{1}\left(i,j\right)+B_{1}\left(i,j\right)+R_{2}\left(i,j\right)+G_{2}\left(i,j\right)+B_{2}\left(i,j\right)\right] \end{array}$$

In this process, ε is considered as a supplementary key for decryption.

### 5.3. Joint Permutation-Diffusion Mechanism with Plaintext-Related Random Pixel Insertion

**Figure 4:**
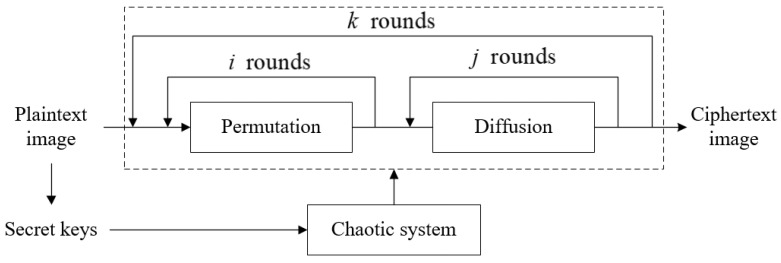
Structure of general image encryption algorithm.

**Figure 5:**
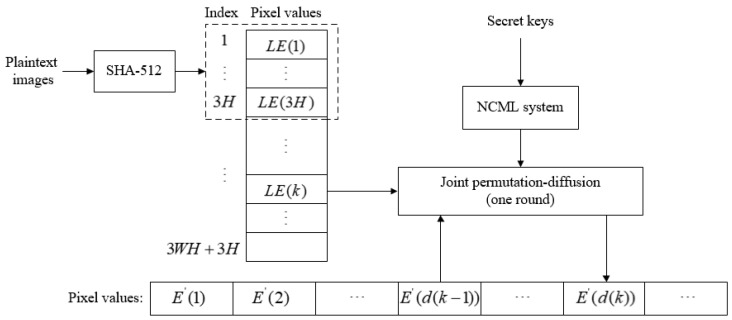
Joint permutation-diffusion mechanism with plaintext-related random pixel insertion.

For the image encryption algorithm with the traditional permutation-diffusion structure shown in Figure 4, there are three main drawbacks: (1) multiple rounds of permutation and diffusion operations improve the level of security but sacrifice efficiency; (2) the secret keys are dependent on the original image and the user could not obtain keys before performing the encryption process; (3) the same ciphertext image is obtained each time when it is applied to the same plaintext image with secret keys, which weakens the robustness of the cryptosystem. Aiming at these shortcomings, a new joint permutation-diffusion mechanism based on the plaintext-related random pixel insertion is designed to acquire the final encryption image E, in which only one-time traversal of the to-be-encrypted sequence is executed [38]. The specific process is shown in Figure 5. The encryption steps are described as follows.

**Step 1**: With the initial keys $x_{1}(1)$, $x_{2}(1)$, δ, and λ, NCML system is iterated $N_{0}+3WH+3H$ times. To eliminate the transient effect, the former $N_{0}$ values are discarded. Then, one can obtain the chaotic sequence $X_{i}$ with length 3WH+3H: $X_{i}=\left\{x_{i}\left(1\right),x_{i}\left(2\right),\ldots ,x_{i}\left(3WH+3H\right)\right\}\left(i=1,2\right)$. Subsequently, the sequence $X_{1}$ is further processed as

(22) $$S=\mathrm{mod}(\mathrm{round}(X_{1}\times 10^{9}),256)$$

Additionally, one could sort sequence $X_{2}$ and record the positions of the corresponding values of the sorted sequence in $X_{2}$ to obtain address sequence d of length 3WH+3H.

**Step 2**: Three matrices r, θ, and φ are converted into a 1D sequence $E_{1}$,

(23) $$\left\{ \begin{array}{l} E_{1}{}_{R}=\mathrm{reshape}\left(r,1,WH\right)\\ E_{1G}=\mathrm{reshape}\left(\theta ,1,WH\right)\\ E_{1B}=\mathrm{reshape}\left(\varphi ,1,WH\right)\\ E_{1}=[E_{R},E_{G},E_{B}] \end{array}\right.$$

Afterwards, one can quantify $E_{1}$ into the range of [0,255],

(24) $$E_{2}=\mathrm{round}\;\left[\frac{255\times \left(E_{1}-\min\left(E_{1}\right)\right)}{\max\left(E_{1}\right)-\min\left(E_{1}\right)}\right]$$

**Step 3:** Generation of random pixel values related to plaintext. Adopting the secret keys $x_{1}(1)$, $x_{2}(1)$, δ and the sum of all the pixels in the original images as the input of hash function SHA-512, a 512-bits hash value V can be obtained. One can randomly select a binary sequence $b=(b_{7}b_{6}\ldots b_{0})$ of length 8 from V for 3H times and convert each binary sequence into decimal integer. Consequently, a plaintext-related random sequence L of length 3H is obtained. Afterwards, sequence L and sequence $E_{2}$ are concatenated into one sequence LE of length 3WH+3H.

**Step 4**: Joint permutation-diffusion mechanism. The first encrypted pixel value $E^{\prime }(1,d(1))$ is randomly selected from sequence L. Then, other encrypted pixel values are obtained by

(25) $$E^{\prime }\left(1,d\left(k\right)\right)=\mathrm{bitxor}\left(\mathrm{mod}\left(\mathrm{bitxor}\left(LE\left(1,k\right),S\left(1,k\right)\right),256\right),E^{\prime }\left(1,d\left(k-1\right)\right)\right)$$

where k=2,…,3WH+3H.

**Step 5**: The final ciphertext E is obtained by

(26) $$E=\mathrm{reshape}(E^{\prime },W,H+1,3)$$

In the proposed joint permutation-diffusion strategy, the plaintext-related random values are randomly inserted in the interim sequence $E^{\prime }$. The value of encrypted sequence $E^{\prime }\left(1,d\left(k\right)\right)$ not only depends on the to-be-encrypted value LE(1,k−1), chaotic value S(1,k), but also is determined by the previous encrypted value $E^{\prime }\left(1,d\left(k-1\right)\right)$, which accords the proposed color image cryptosystem a high level of security, as elaborated in the later section.

### 5.4. Double-Color-Image Decryption Algorithm

**Figure 6:**
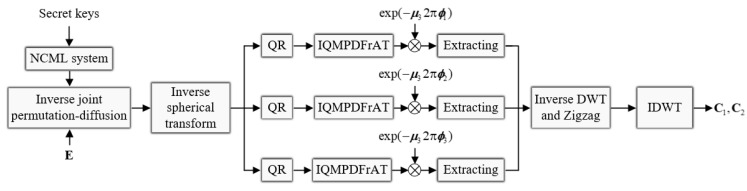
Double-color-image decryption algorithm.

Since the proposed double-color-image encryption algorithm is symmetric, those who know the whole keys can decrypt the ciphertext with the reverse encryption process described in Section 5.2. The decryption process is exhibited in Figure 6. Particularly, the inverse decryption process of joint permutation-diffusion strategy is

(27) $$\left\{ \begin{array}{l} LE(1,1)=E^{\prime }(1,d(1))\\ LE\left(1,k\right)=\mathrm{bitxor}\left(S\left(k,1\right),\mathrm{mod}\left(\mathrm{bitxor}\left(E^{\prime }\left(1,d\left(k\right)\right),E^{\prime }\left(1,d\left(k-1\right)\right)\right)+256,256\right)\right) \end{array}\right.$$

where k=2,…,3WH+3H. The inverse transform for Equation (20) is

(28) $$\left\{ \begin{array}{l} B_{1}\left(u,v\right)=r\left(u,v\right)\sin\frac{\theta \left(u,v\right)}{\varepsilon }\cos\frac{\varphi \left(u,v\right)}{\varepsilon }\\ B_{2}\left(u,v\right)=r\left(u,v\right)\sin\frac{\theta \left(u,v\right)}{\varepsilon }\sin\frac{\varphi \left(u,v\right)}{\varepsilon }\\ B_{3}\left(u,v\right)=r\left(u,v\right)\cos\frac{\theta \left(u,v\right)}{\varepsilon } \end{array}\right.$$

After performing the inverse QMPDFrATs and inverse phase mask modulations, one can extract the four parts of each resulting quaternion signals, respectively. Finally, the decryption images can be retrieved through the inverse compression process and inverse DWT operation.

## 6. Simulation Results and Security Analyses

### 6.1. Encryption and Decryption Results

**Figure 7:**
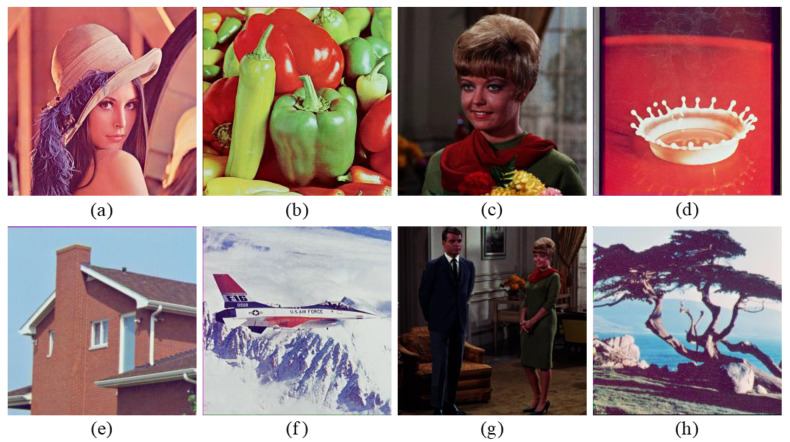
Original images: (**a**) “Lena”, (**b**) “Peppers”, (**c**) “Female”, (**d**) “Milkdrop”, (**e**) “House”, (**f**) “Airplane”, (**g**) “Couple”, (**h**) “Tree”.

**Figure 8:**
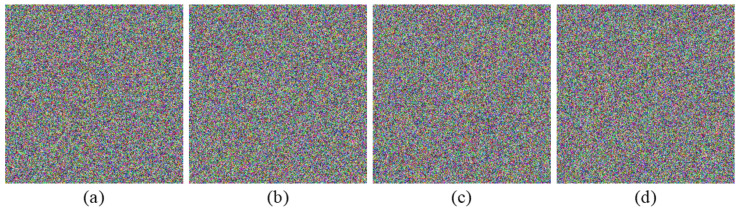
Ciphertext images: (**a**) “Lena-Peppers”, (**b**) “Female-Milkdrop”, (**c**) “House-Airplane”, (**d**) “Couple-Tree”.

**Figure 9:**
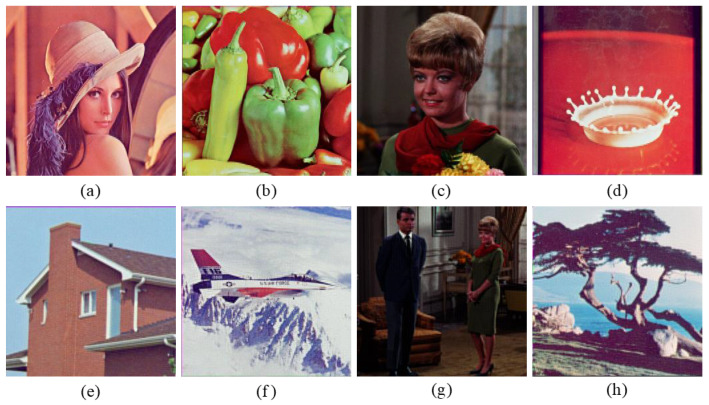
Decryption images: (**a**) “Lena”, (**b**) “Peppers”, (**c**) “Female”, (**d**) “Milkdrop”, (**e**) “House”, (**f**) “Airplane”, (**g**) “Couple”, (**h**) “Tree”.

To verify the feasibility of the proposed encryption algorithm, four groups of color images of size 256×256 shown in Figure 7 are selected from the USC-SIPI image database to be tested [39]. The secret keys $M_{1}$ and $M_{2}$ are arbitrarily taken as 25 and 29, respectively. The pure quaternions $\mu_{1}$, $\mu_{2}$, $\mu_{3}$, and $\mu_{4}$ are set as i, j, k, and $\left(i+j+k\right)/\sqrt{3}$, respectively. The $M_{i}$-dimensional parameter vector $\eta_{i}\left(i=1,2\right)$ is random real vector whose values are independent and uniformly distributed in [0,100]. The fractional orders α and β are randomly given as 0.4697 and 0.4023, respectively. The initial values and control parameters of the NCML system are chosen arbitrarily as: $x_{1}(1)=0.4728$, $x_{2}(1)=0.3977$, δ=0.2635, λ=3.9864, respectively. Figure 8 and Figure 9 show the encryption and the decryption results, respectively. To measure the quality of restored image, two image quality assessment criteria are considered, i.e.,

(1) Peak Signal-to-Noise Ratio (PSNR) is
  (29) $$\mathrm{PSNR}=10\log\frac{W\times H\times 255^{2}}{\sum_{m=1}^{W}\sum_{n=1}^{H}{\left[C(m,n)-D(m,n)\right]}^{2}}$$
  where C(m,n) and D(m,n) represent the pixel values of each color component of the original color image and the decryption one, respectively.
(2) Structural similarity (SSIM) index [40] is
  (30) $$\mathrm{SSIM}\left(x,y\right)=\frac{\left(2\mu_{x}\mu_{y}+c_{1}\right)\left(2\sigma_{xy}+c_{2}\right)}{\left(\mu_{x}^{2}+\mu_{y}^{2}+c_{1}\right)\left(\sigma_{x}^{2}+\sigma_{y}^{2}+c_{2}\right)}$$
  where x and y are the windows of two images with size m×m, $\mu_{x}$ and $\mu_{y}$ denote the average values of x and y, $\sigma_{x}^{2}$ and $\sigma_{y}^{2}$ are variances of x and y, respectively, $\sigma_{xy}$ is the covariance between x and y. $c_{1}={\left(k_{1}L\right)}^{2}$, $c_{2}={\left(k_{2}L\right)}^{2}$, $k_{1}=0.01$, $k_{2}=0.03$, L is the gray level of the plaintext image. The greater SSIM means the better recovery of image.

The PSNR values and the mean SSIM (MSSIM) values for different images are collected in Table 3. It can be seen from Figure 8 and Figure 9 and Table 3 that the ciphertext images cannot reveal the information of the original images and the decryption images achieve good reconstruction quality.

### 6.2. Decryption Quality Evaluation

In the conventional DWT-based image compression and encryption methods, to achieve the purpose of compression, only the low-frequency part of the original image is utilized for encryption and the high-frequency parts are discarded, which affects the decryption quality of the image [41,42]. In this paper, to achieve compression and improve the quality of image reconstruction simultaneously, five methods shown in Table 4 are designed to flexibly select the high-frequency parts of the original images.

**Figure 10:**
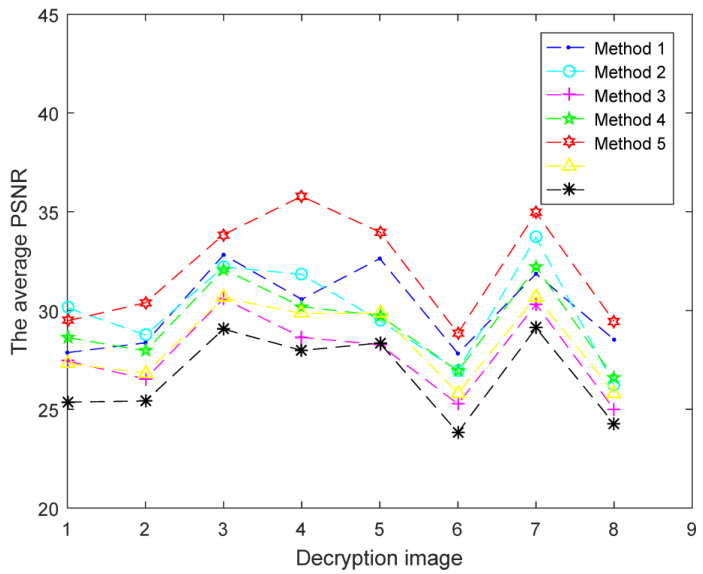
PSNR values with different methods: the eight points on the abscissa denote decryption images “Lena”, “Peppers”, “Female”, “Milkdrop”, “House”, “Airplane”, “Couple”, and “Tree”, respectively. Yellow [29], Black [31].

**Figure 11:**
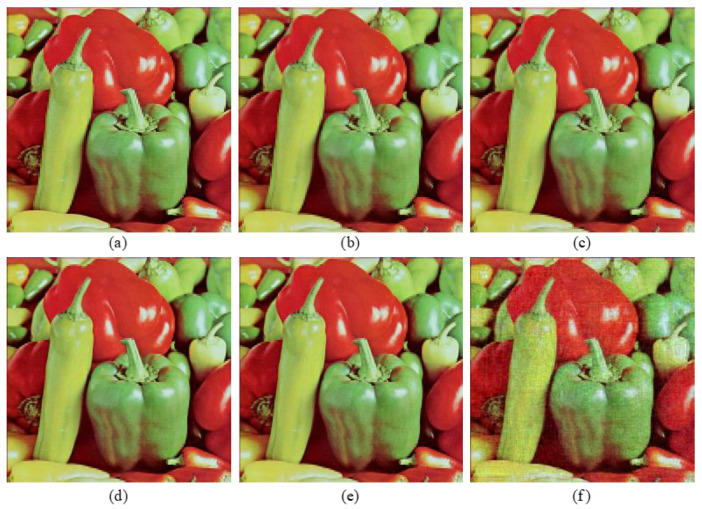
Decryption results: (**a**–**f**) decryption images “Peppers” with methods 1, 2, 3, 4, 5, and method in ref. [31], respectively.

**Figure 12:**
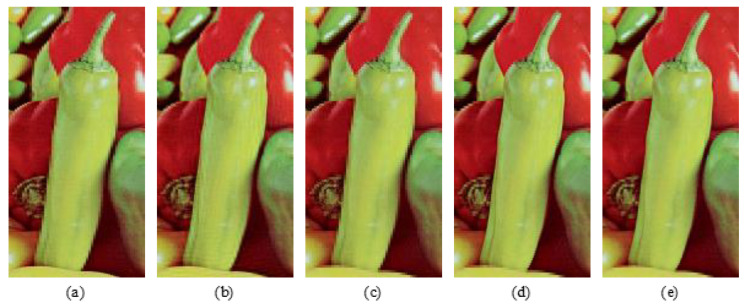
(**a**–**e**) Details of the decryption image “Peppers” with methods 1, 2, 3, 4, and 5, respectively.

Simulations are conducted with the five above-mentioned methods. The corresponding PSNR values of different decryption images are depicted in Figure 10. Decryption images with the proposed five methods have relatively higher reconstruction quality than those in [29,31], since both the low-frequency parts and the high-frequency parts of original images are reserved to be encrypted. As an example, decryption images “Peppers” with five methods are shown in Figure 11. Corresponding selected details of decryption “Peppers” are exhibited in Figure 12. From Figure 10, Figure 11 and Figure 12, although the PSNR value of the decryption image with method 1 (2, 3) is acceptable, the details of the corresponding decryption image are distorted obviously, for only one of the three high-frequency parts associated with the original RGB components are reserved to be encrypted. For method 4, the selected high-frequency parts ($LH_{1}$, $HL_{1}$, $HH_{1}$) of each RGB component are different and the decryption images contain all these three high-frequency parts as possible, which leads to the decryption images not only achieving similar decryption quality with method 1 (2, 3), but also reducing the undesirable distortion effect of the detail part to a certain extent. For method 5, the main information of all the three high-frequency parts of every RGB component are reserved via the DWT and Zigzag operation, which can make the decryption images achieve higher visual quality and relatively higher reconstruction quality than method 4. Based on the above discussion, the adoption of method 5 as the feature fusion of the high-frequency parts of the original images is more helpful for improving the reconstruction quality of decryption image.

### 6.3. Statistical Analyses

#### 6.3.1. Histogram Analysis

**Figure 13:**
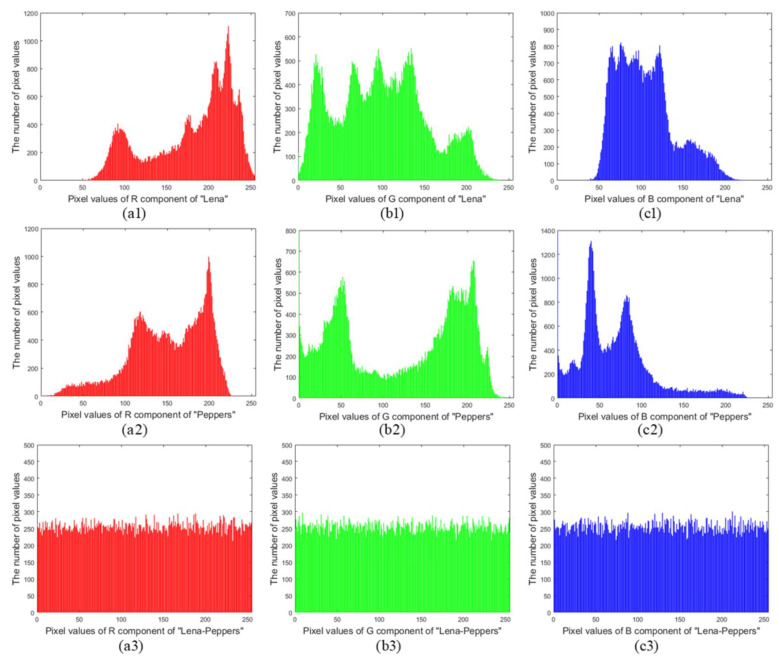
Histograms: (**a1**–**c1**) R, G, B components of image “Lena”, respectively. (**a2**–**c2**) R, G, B components of image “Peppers”, respectively. (**a3**–**c3**) R, G, B components of encryption image “Lena-Peppers”, respectively.

Histograms play an important role in statistical analyses. Figure 13(a1–c1,a2–c2) are the histograms of RGB components of original “Lena” and “Peppers”, respectively. Figure 13(a3–c3) are the histograms of RGB components of encryption image, respectively. In the encryption process, the proposed QMPDFrAT is performed on the compressed image, which causes the histograms of the intermediate results have a similar distribution. Afterward, the proposed joint permutation-diffusion operation can make the pixel values of intermediate results distributed uniformly among the range of 0–255. From Figure 13, the histograms of RGB components of original color images “Lena” and “Peppers” are quite different while those of RGB components of the encryption image show similarity and uniform distribution. In addition, the chi-square $\left(\chi^{2}\right)$ test is adopted to numerically measure the uniformity of the histogram of ciphertext [43], i.e.,

(31) $$\chi^{2}=\sum_{L=0}^{255}\frac{{\left(o_{L}-e_{L}\right)}^{2}}{e_{L}}$$

where $o_{L}$ is the observed number of the *L*-th gray level and $e_{L}$ is the expected number of the *L*-th gray level. Table 5 gives the results of the chi-square test for the RGB components of the encryption image under different input images. From Table 5, the $\chi^{2}$-values of encrypted RGB components are under the critical values with 1% and 5% level of significance, which indicates that the proposed encryption algorithm can withstand the histogram attack.

#### 6.3.2. Correlation Analysis

**Figure 14:**
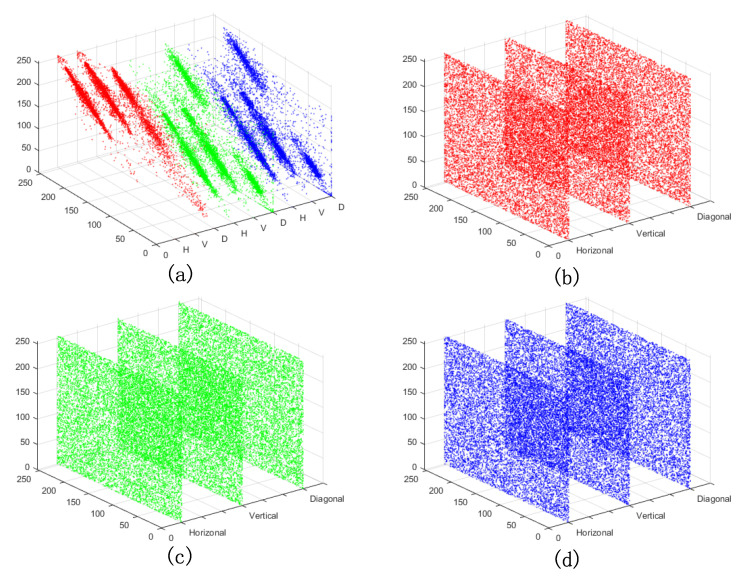
Correlation distributions of adjacent pixels in the horizontal, vertical, diagonal directions: (**a**) distribution of original color image “Lena”; (**b**–**d**) distributions of red, green, and blue components of encryption image “Lena−Peppers”, respectively.

A total of 12,000 pairs of adjacent pixels in the horizontal, vertical, and diagonal directions are chosen randomly from the original color image “Lena” and the corresponding encryption image. Their correlation distributions are displayed in Figure 14. In Figure 14, the correlation distributions of the three color channels of the original image “Lena” are linear and strongly correlated, while those of the corresponding three color channels of the encryption image are almost uniform. Moreover, to evaluate this feature numerically, the correlation coefficients of the selected 12,000 pairs of adjacent pixels in three directions are calculated, as shown in Table 6. The correlation coefficients in the original color images are close to 1, while those in the encryption images are near 0. The results suggest that the proposed algorithm can reduce the correlation in original images significantly. Therefore, the statistical analysis attack is impracticable for the proposed double−color−image encryption algorithm.

#### 6.3.3. Information Entropy Analysis

Information entropy H(m) can reflect the degree of randomness and the unpredictability of a random event m, i.e.,

(32) $$H\left(m\right)=\sum_{i=0}^{2^{N}-1}p\left(m_{i}\right)\log\frac{1}{p\left(m_{i}\right)}$$

where $p\left(m_{i}\right)$ is the occurrence probability of the random event $m_{i}$. Theoretically, the value of H(m) for an encryption image with 256−level gray is 8 bits when all gray levels obey the uniform probability distribution. In our double−color−image encryption algorithm, the ciphertext image is obtained by the QMPDFrAT and chaos-based joint permutation−diffusion, which makes encrypted pixel values randomly distributed as much as possible. The information entropies of the RGB components in the final encryption image under different input test images are shown in Table 7. One can see that the information entropies are extremely close to 8 bits. Therefore, the proposed double-color-image encryption algorithm can resist the information entropy analysis attack.

### 6.4. Sensibility Analyses

#### 6.4.1. Key Sensitivity Analysis

**Figure 15:**
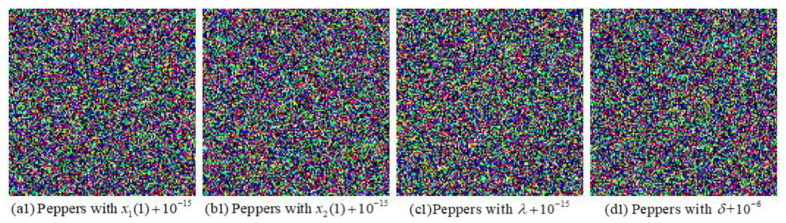
Decryption image “Peppers” with adjusted keys: (**a1**) $x_{1}{\left(1\right)}^{\prime }=x_{1}(1)+10^{-15}$, (**b1**) $x_{2}{\left(1\right)}^{\prime }=x_{2}(1)+10^{-15}$, (**c1**) $\lambda^{\prime }=\lambda +10^{-15}$, (**d1**) $\delta^{\prime }=\delta +10^{-6}$.

**Figure 16:**
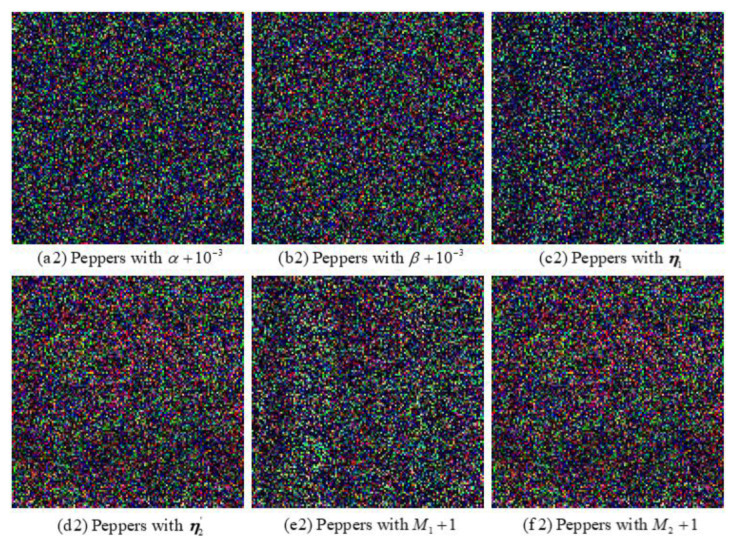
Decryption image “Peppers” with adjusted keys: (**a2**) $\alpha^{\prime }=\alpha +10^{-3}$, (**b2**) $\beta^{\prime }=\beta +10^{-3}$, (**c2**) $\eta_{1}^{\prime }$, (**d2**) $\eta_{2}^{\prime }$ (**e2**) $M_{1}^{\prime }=M_{1}+1$, (**f2**) $M_{2}^{\prime }=M_{2}+1$.

To inspect the sensitivity of the proposed algorithm, a set of tests are performed by decrypting the ciphertext image with a tiny perturbation in the correct encryption key. Figure 15 and Figure 16 exhibit the decryption image “Peppers” when one of the initial keys has a tiny deviation while all the other keys are correct, respectively. Figure 16 shows the decryption image “Peppers” decrypted with wrong keys $\alpha^{\prime }=\alpha +10^{-3}$, $\beta^{\prime }=\beta +10^{-3}$, $M_{1}^{\prime }=M_{1}+1$, $M_{2}^{\prime }=M_{2}+1$, randomly generated real vectors $\eta_{1}^{\prime }$ and $\eta_{2}^{\prime }$, respectively. The decryption results indicate that these images cannot reveal any serviceable information and the proposed image encryption algorithm is sensitive to the above-mentioned keys.

#### 6.4.2. Key Space Analysis

Simulations show that the secret keys $\theta_{1}$, $\theta_{2}$, and ε are not sensitive enough, thus they are considered supplementary keys. From the sensitivity analysis in Section 6.4.1, the precision of the keys $x_{1}(1)$, $x_{2}(1)$, and λ is up to $10^{-15}$. The deviation of control parameter δ is about $10^{-6}$. The key space for fractional order α (β) is $10^{-3}$. Therefore, the total key space of the proposed algorithm is at least $10^{57}$, which is greater than $2^{189}$. It indicates that the key space of the proposed encryption algorithm is large enough to resist the brute-force attack.

#### 6.4.3. Differential Attack Analysis

Two common indicators, i.e., NPCR (number of pixel change rate) and UACI (unified average changing intensity) are introduced to evaluate the ability of the proposed algorithm to resist differential attack. These two indicators can be computed, respectively, as [11]

(33) $${\mathrm{NPCR}}_{R,G,B}=\sum_{i,j}\frac{D_{R,G,B}\left(i,j\right)}{W\times H}\times 100\%$$

(34) $${\mathrm{UACI}}_{R,G,B}=\sum_{i,j}\frac{\left|E_{R,G,B}^{\prime }\left(i,j\right)-E_{R,G,B}\left(i,j\right)\right|}{255\times W\times H}\times 100\%$$

(35) $$D_{R,G,B}\left(i,j\right)=\{\begin{array}{c} 0,E_{R,G,B}^{\prime }\left(i,j\right)=E_{R,G,B}\left(i,j\right)\\ 1,E_{R,G,B}^{\prime }\left(i,j\right)\neq E_{R,G,B}\left(i,j\right) \end{array}$$

where $E_{R,G,B}$ and $E_{R,G,B}^{\prime }$ are the ciphertext images without and with only one pixel altered in the plaintext images, respectively. In these experiments, 10 pixels of different positions in each plaintext image are randomly selected and only one pixel is changed each time. In the diffusion process, the plaintext-related random values are randomly inserted into the to-be-encrypted sequence and the encrypted values are determined by the chaotic values and their previous ciphered values, both of which make the proposed cryptosystem sensitive to plaintext images. The average NPCR values and the average UACI values for the two ciphertext images are tabulated in Table 8. It shows that the proposed encryption algorithm could resist differential attack, since the values of NPCR and UACI are close to their theoretical values.

### 6.5. Robustness against Noise Analysis and Data Loss Attack

**Figure 17:**
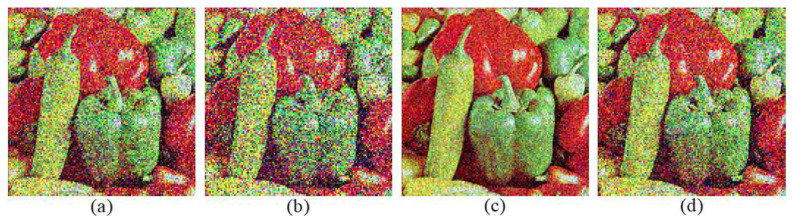
Decryption images “Peppers” with different noise attacks: Gaussian noise with intensity k (**a**) k=0.25, (**b**) k=0.75, Salt and Pepper noise with the density of noise distribution (**c**) 0.1 (**d**) 0.5.

**Figure 18:**
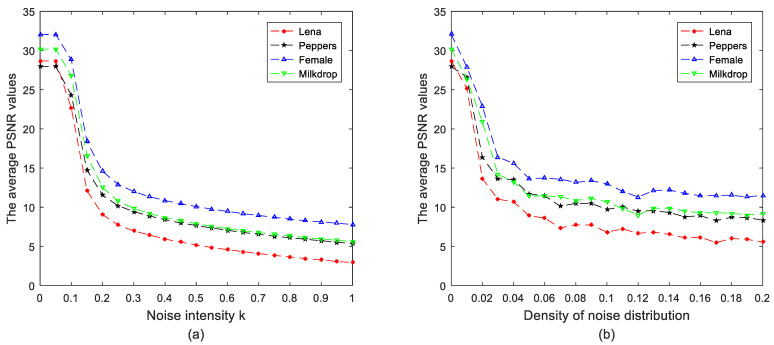
Average PSNR value versus noise parameter: (**a**) Gaussian noise, (**b**) Salt and Pepper noise.

**Figure 19:**
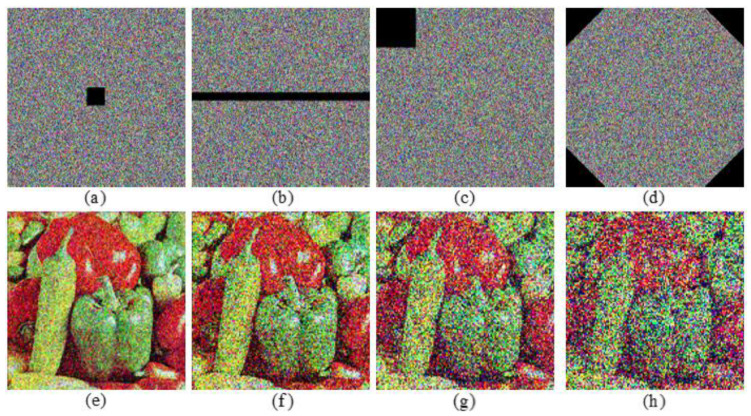
Results of data loss attack: (**a**–**d**) are encryption images with 1%, 2.5%, 5%, 10% data loss, respectively; (**e**–**h**) are the corresponding decryption images “Peppers”.

Assume that the encryption image is polluted by the additive Gaussian noise and Salt and Pepper noise during transmission. Decryption results of image “Peppers” with these two types of noises added to the ciphertext are displayed in Figure 17. Although the quality of decryption images decreases with the increase of noise parameter, the decryption images are still identifiable. It indicates that the proposed color image encryption algorithm could resist the noise attack to a certain extent. Figure 18 shows the PSNR values of different decryption images with the increase of noise parameter, which further supports our conclusion. To analyze the robustness of the proposed algorithm against data loss attack, the ciphertext image is assumed to be cropped to a limited degree. Simulation results are exhibited in Figure 19. It can be noted that the main information of the decryption image can still be recognized since the main information of the plaintext images is randomly distributed over the whole ciphertext image by the proposed QMPDFrAT and the joint permutation-diffusion mechanism. Therefore, the proposed algorithm can withstand data loss attack to a limited degree.

### 6.6. Robustness of the Proposed Algorithm against Four Typical Attacks

Among the four potential attacks including ciphertext-only attack, known-plaintext attack, chosen-ciphertext attack, and chosen-plaintext attack, the chosen-plaintext attack is considered as the most powerful one. In the cryptanalysis, if the cryptosystem is immune to the chosen-plaintext attack, it will be able to withstand other three attacks [12].

**Figure 20:**
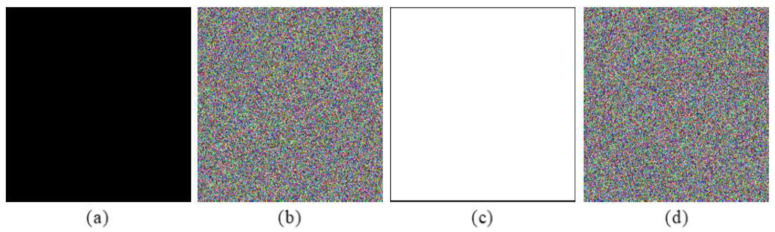
Encryption results of black and white images: (**a**) black image, (**b**) encryption black image, (**c**) white image, (**d**) encryption white image.

Under the chosen-plaintext attack, attackers may deduce the secret keys by a pair of the corresponding plaintext and ciphertext images. In our algorithm, the deficiency caused by the linear transform system is eliminated by a nonlinear spherical transform. The current encrypted pixel value is associated with the plaintext-related values and the previous ciphered value, which contributes to the high sensitivity for the plaintext images. On the other hand, the plaintext-related values are obtained in a random way, which enables the proposed algorithm to generate a completely different encrypted images each time when it is applied to the same original images with the same secret keys. In addition, some attackers may deduce the secret keys by analyzing the special images, such as all black and all white images [44]. To analyze this situation, double black images and double white images are considered as the inputs of the proposed cryptosystem, respectively. Figure 20 shows one of the double special images and their corresponding encryption images. As it is shown from the simulation results, the ciphertext images of these two special images are all noise-like. Therefore, the designed double-color-image encryption algorithm has a strong ability to resist the chosen-plaintext attack and the other three potential attacks.

### 6.7. Time Analysis

**Figure 21:**
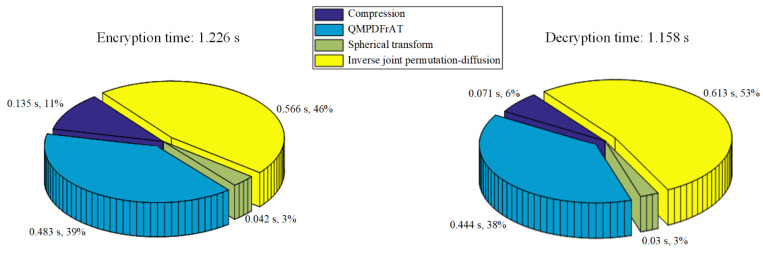
(**a**) Encryption time and time consumption percentage of each part; (**b**) decryption time and time consumption percentage of each part.

Execution time is a significant consideration in image encryption and decryption processes. The encryption and decryption time of the proposed cryptosystem and similar algorithms in refs. [11,29,31,38] is shown in Table 9. Simulations with the same number of input images are conducted under MATLAB (R2016a) on a personal computer with Intel (R) Core (TM) i7−3537 U CPU @2.00 GHz, 4GB RAM running Windows 10. In ref. [11], the keystreams utilized in encryption and decryption processes are generated by iterating the 6D hyperchaotic system, which takes too much time. In refs. [29,31], the compression and encryption are realized efficiently by combining CS with joint low-dimensional chaotic system. However, the decryption process is time-consuming as it takes too much time to reconstruct the original signal. Since the whole encryption process is executed in the spatial domain, the security of the encryption algorithm in ref. [38] is guaranteed by the complex permutation and diffusion operations, which leads to relatively longer encryption and decryption time. In our algorithm, the time-consuming parts include double-color-image compression, three times QMPDFrATs, a spherical transform, and one-time joint permutation-diffusion operation. QMPDFrAT was pointed out to be efficient in Section 3. Only one-time traversal of the to-be-encrypted sequence allows the permutation-diffusion process to take relatively shorter encryption and decryption time. Figure 21a,b shows the encryption time and the decryption time of each part, respectively. As observed from Figure 21, the encryption and decryption time is acceptable. Therefore, the proposed image compression-encryption algorithm is feasible in real-time cryptosystem.

## 7. Conclusions

The quaternion multiple parameter discrete fractional angular transform is firstly defined. The analysis shows that the proposed quaternion multiple parameter discrete fractional angular transform is a suitable tool for image encryption. Based on this transform, a new double-color-image compression-encryption algorithm with a spatiotemporal chaotic system is obtained. Sub-bands of original images based on quaternion representation are encrypted with quaternion multiple parameter discrete fractional angular transform and the intermediate results are constructed into three new matrices with the same size of plaintext images, which avoids the additional data extension that many transform-based methods yield. The spherical transform, as a nonlinear operation, is introduced to nonlinearly make the three transform results interact. A new joint permutation-diffusion mechanism with plaintext-related random pixel insertion is developed to enhance the security of cryptosystem and reduce encryption time simultaneously. The simulation results show that the proposed algorithm has better reconstruction effects than some similar compression-encryption algorithms. The security performance evaluation demonstrates that the proposed color image encryption algorithm has a large key space, high key sensitivity, and can withstand statistical analyses attack, differential attack, noise attack, occlusion attack, known-plaintext attack, and chosen-plaintext attack.

## Figures and Tables

**Table 1:** Comparison results under the same compression ratio.

Spare and Reconstruction Algorithms	DWT + OMP	$\mathrm{DWT}+{\mathrm{SL}}_{0}$	DWT + IDWT
Decryption time (s)	3.5582	3.6933	0.0369
PSNR	20.3441	20.5292	30.5270

**Table 2:** Comparison of four quaternion transforms.

Transform	FRQFT [35]	QDFRNT [36]	MPFrQFT [19]	Proposed QMPDFrAT
Performance	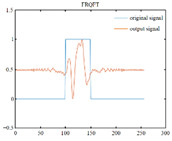	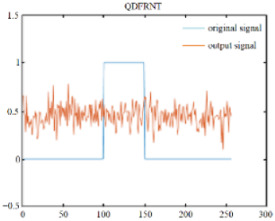	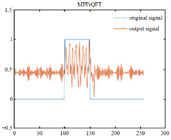	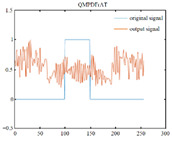
Secret keys	α,μ	α,μ	$\alpha ,\mu ,M_{1},\eta_{1}$	$\alpha ,\theta ,\mu ,M_{1},\eta_{1}$
Calculation time (s) of eigenvector (N = 256)	0.014513	0.021912	0.014513	0.004462

**Table 3:** PSNR and MSSIM values of decryption images.

Decryption Image	PSNR (dB)				MSSIM
	R	G	B	Average	
“Lena”	30.8783	28.1759	29.3766	29.4769	0.9801
“Peppers”	31.1626	29.1326	30.8829	30.3927	0.9903
“Female”	34.9543	34.1980	32.3790	33.8437	0.9925
“Milkdrop”	41.5283	32.1835	33.6339	35.7819	0.9957
“House”	34.6128	34.6335	32.6833	33.9765	0.9946
“Airplane”	28.3353	27.4659	30.7483	28.8499	0.9787
“Couple”	34.8817	35.7362	34.2417	34.9532	0.9904
“Tree”	30.6282	28.3096	29.3898	29.4425	0.9842

**Table 4:** Five methods for selecting high-frequency sub-bands.

Method	6 High-Frequency Sub-Bands
Method 1	$LH_{1}^{R_{1},G_{1},B_{1},R_{2},G_{2},B_{2}}$
Method 2	$HL_{1}^{R_{1},G_{1},B_{1},R_{2},G_{2},B_{2}}$
Method 3	$HH_{1}^{R_{1},G_{1},B_{1},R_{2},G_{2},B_{2}}$
Method 4	$LH_{1}^{R_{1},R_{2}},HL_{1}^{G_{1},G_{2}},HH_{1}^{B_{1},B_{2}}$
Method 5	$\begin{array}{l} \left(LH_{1}^{R_{1},R_{2}}+HL_{1}^{R_{1},R_{2}}+HH_{1}^{R_{1},R_{2}}\right)\underset{\to }{\mathrm{DWT}\&\mathrm{Zigzag}}C_{1}^{R_{1},R_{2}}\\ \left(LH_{1}^{G_{1},G_{2}}+HL_{1}^{G_{1},G_{2}}+HH_{1}^{G_{1},G_{2}}\right)\underset{\to }{\mathrm{DWT}\&\mathrm{Zigzag}}C_{1}^{G_{1},G_{2}}\\ \left(LH_{1}^{B_{1},B_{2}}+HL_{1}^{B_{1},B_{2}}+HH_{1}^{B_{1},B_{2}}\right)\underset{\to }{\mathrm{DWT}\&\mathrm{Zigzag}}C_{1}^{B_{1},B_{2}} \end{array}$

**Table 5:** Results of chi-square test.

Image	*χ*^2^-Value			Critical Value	
	R	G	B	1% Probability	5% Probability
“Lena-Peppers”	244.9391	227.9453	260.7734	310.457	293.2478
“Female-Milkdrop”	239.0859	243.7813	249.9844		
“House-Airplane”	267.0547	223.8281	261.0781		
“Couple-Tree”	243.7188	249.2656	191.0938		

**Table 6:** Correlation coefficients of adjacent pixels.

Scheme	Image		Horizontal Direction	Vertical Direction	Diagonal Direction
Proposed scheme	“Lena”	R	0.9662	0.9355	0.9056
		G	0.9459	0.9047	0.8735
		B	0.8931	0.8662	0.8314
	“Peppers”	R	0.9507	0.9453	0.9069
		G	0.9652	0.9571	0.9307
		B	0.9451	0.9356	0.9028
	“Female”	R	0.9553	0.9716	0.9401
		G	0.9653	0.9722	0.9547
		B	0.9493	0.9607	0.9360
	“Milkdrop”	R	0.9947	0.9824	0.9809
		G	0.9710	0.9572	0.9418
		B	0.9542	0.9567	0.9182
	“Lena−Peppers”	R	−0.0013	−0.0111	0.0046
		G	0.0135	0.0064	−0.0241
		B	0.0179	0.0131	0.0023
	“Female−Milkdrop”	R	0.0016	0.0044	−0.0013
		G	−0.0120	0.0095	0.0056
		B	0.0017	0.0094	0.0141
Ref. [11]	“Lena”	R	−0.0027	−0.0131	−0.0032
		G	0.0073	0.0178	0.0127
		B	0.0012	−0.0140	0.0123
Ref. [31]	“Lena”	R	0.0847	0.0501	0.0356
		G	0.0707	−0.0318	0.0831
		B	0.1394	−0.0133	0.1065
Ref. [38]	“Lena”	R	0.0025	0.0047	0.0021
		G	0.0019	0.0127	0.0037
		B	−0.0032	0.0101	0.0087

**Table 7:** Results of information entropy (dB) of encryption color images.

Algorithm	Encryption Image	R	G	B
Proposed algorithm	“Lena−Peppers”	7.9970	7.9974	7.9976
	“Female−Milkdrop”	7.9973	7.9974	7.9971
	“House−Airplane”	7.9970	7.9976	7.9975
	“Couple−Tree”	7.9973	7.9973	7.9979
Ref. [11]	“Lena”	7.9915	7.9912	7.9909
Ref. [30]	“Lena”	7.3488	7.4637	7.3369
Ref. [31]	“Lena”	7.2496	7.3555	7.2855
Ref. [38]	“Lena”	7.9990	7.9989	7.9992

**Table 8:** Results of average NPCR and UACI values for different color images.

Image	NPCR (%)			UACI (%)		
	Red	Green	Blue	Red	Green	Blue
“Lena”	99.6429	99.6628	99.6261	33.4440	33.4876	33.4167
“Peppers”	99.6325	99.6118	99.6253	33.4530	33.4729	33.4237
“Female”	99.6536	99.6332	99.6379	33.4521	33.4136	33.4377
“Milkdrop”	99.6045	99.5992	99.6210	33.3561	33.4129	33.4459
“Lena” in Ref. [11]	99.6101	99.6136	99.6141	33.4695	33.4292	33.4665
“Lena” in Ref. [31]	99.6258	99.6366	99.5997	33.3894	33.3944	33.4859
“Lena” in Ref. [38]	99.6103	99.9098	99.6089	33.4655	33.4652	33.4591

**Table 9:** Encryption and decryption time (s).

Time	Proposed Scheme	Ref. [11]	Ref. [29]	Ref. [31]	Ref. [38]
Encryption time	1.2271	4.0821	0.8574	0.9139	1.8094
Decryption time	1.1579	4.2116	4.6658	4.5348	1.9225

## Data Availability

Not applicable.
